# Aging features of the migratory locust at physiological and transcriptional levels

**DOI:** 10.1186/s12864-021-07585-3

**Published:** 2021-04-10

**Authors:** Siyuan Guo, Pengcheng Yang, Bo Liang, Feng Zhou, Li Hou, Le Kang, Xianhui Wang

**Affiliations:** 1 State Key Laboratory of Integrated Management of Pest Insects and Rodents, Institute of Zoology, Chinese Academy of Sciences, Beijing, 100101 China; 2 CAS Center for Excellence in Biotic Interactions, University of Chinese Academy of Sciences, Beijing, 100049 China; 3 Beijing Institutes of Life Science, Chinese Academy of Sciences, Beijing, 100101 China

**Keywords:** Aging, Non-*Drosophila* insect, Systematic assessments, Organ specificity, Transcriptomics, RNA interference

## Abstract

**Background:**

Non-*Drosophila* insects provide diverse aging types and important complementary systems for studies of aging biology. However, little attention has been paid to the special roles of non-*Drosophila* insects in aging research. Here, the aging-related features of the migratory locust, *Locusta migratoria*, were determined at the physiological, cellular, and transcriptional levels.

**Results:**

In physiological assessments, the flight performance and sperm state of locusts displayed clear aging-related decline in male adults. Transcriptional analyses demonstrated locusts have similar aging-related genes with model species. However, different from those of *Drosophila* and mammals, the organ-specific aging transcriptional features of locusts were characterized by intensive expression changes in flight muscle and fat body and little transcriptional changes in brain. The predominant transcriptional characteristics of flight muscle and fat body aging were changes in expression of mitochondrion-related genes and detoxification and phagocytosis genes, respectively. Cellular assessments revealed the incidence of mitochondrial abnormalities significantly increased in aged flight muscle, and apoptotic signals and nuclear abnormalities were enhanced in aged fat body but not in brain. In addition, some well-known aging genes and locust aging-related genes (i.e., *IAP1*, *PGRP-SA*, and *LIPT1*), whose roles in aging regulation were rarely reported, were demonstrated to affect lifespan, metabolism, and flight ability of locusts after RNAi.

**Conclusion:**

This study revealed multi-level aging signatures of locust, thus laying a foundation for further investigation of aging mechanisms in this famous insect in the future.

**Supplementary Information:**

The online version contains supplementary material available at 10.1186/s12864-021-07585-3.

## Background

Aging is the greatest risk factor of most chronic pathological conditions [1]. However, due to the extraordinary complexity of aging, a lack of consensus remains even on the most fundamental questions in this field [2]. Developing novel aging systems is of great value for improving the understanding of aging biology [3]. As the most diverse group of animals, insects have several characteristics which are of great value for aging study. For examples, the extreme intraspecific variations in the lifespan of social insects offer an opportunity to study how aging is differentially regulated by social factors; the insect diapause, as a “non-aging” state, provides an opportunity to study methods of aging intervention [4].

Locusts are regarded as a classic model species for research on insect morphology, behavior, and physiology [5–8]. Because of their large body size and polyphenisms, locusts have recently been proposed as a study model for aging biology. Gordon et al. [9] revealed sex-dependent hearing ability decline in old desert locusts, characterized by progressive altered tympanal membrane displacement, decreased neurophysiological response, and prolonged latency for neurobiological response from 2 weeks after eclosion. The aging of desert locusts is also accompanied with an increased diversity of gut microbiota at 4 weeks after eclosion and a slow rate of thickening of the tibia 3 weeks after eclosion [10, 11]. Moreover, locusts display density-dependent phenotypic plasticity, wherein gregarious locusts have a much shorter lifespan than solitary ones [12]. The lifespan divergences between two phases are determined by population density at early nymphal stages [12]. Although some progress has been made in understanding locust aging, the aging features of locusts have not been systematically characterized until now.

In multicellular organisms, various organs perform distinct but coordinated physiological roles to ensure proper functions. Different organs display specific aging-related deteriorated rates and resilience [13, 14]. For instance, the human cardiovascular system appears to suffer from an aging-dependent functional decline more rapidly than the gastrointestinal system [14]. Studying organ-specific aging is important because signal events occurring in a single organ often drive the aging processes of other organs and whole organisms through inter-organ communication [15]. The aging signs of locusts occur in different organs at specific time points, indicating that locusts may also display organ-specific aging [9–11], although direct evidence is still lacking.

Aging is accompanied by remarkably transcriptional changes across various organs from worms to humans [16–21]. Aging-related transcriptional changes display species and organ specificities [22]. For example, the transcriptional profiles of human skin are extraordinarily susceptible to aging [23], and *Drosophila* muscle displayed larger transcriptional changes than other organs upon aging [21]. Noteworthy, these organ-specific gene expression shifts are not only responsible for aging processes of individual organs, but also potentially contribute to systemic aging by affecting the corresponding protein levels in plasma [24]. Thus, organ-level transcriptional analysis is the key to in-depth understanding of aging processes in given species.

The migratory locust, *Locusta migratoria*, is a widely distributed locust species. Advances in genomic information and genetic tools have allowed for systematic assessment of the aging characteristics of the migratory locusts [25, 26]. In the present study, several physiological and cellular aging phenotypes were assessed in gregarious locusts. Organ-level transcriptional profiles along aging were obtained to analyze the transcriptional similarity across species and organ-specific transcriptional signatures. The functional roles of several aging-related genes were also verified using RNAi. This study unraveled the largely conserved but partially specific aging phenotypes in locusts, thus providing a basis for further investigation of aging in this non-*Drosophila* insect.

## Results

### Aging-dependent physiological phenotypes in locusts

**Fig. 1:**
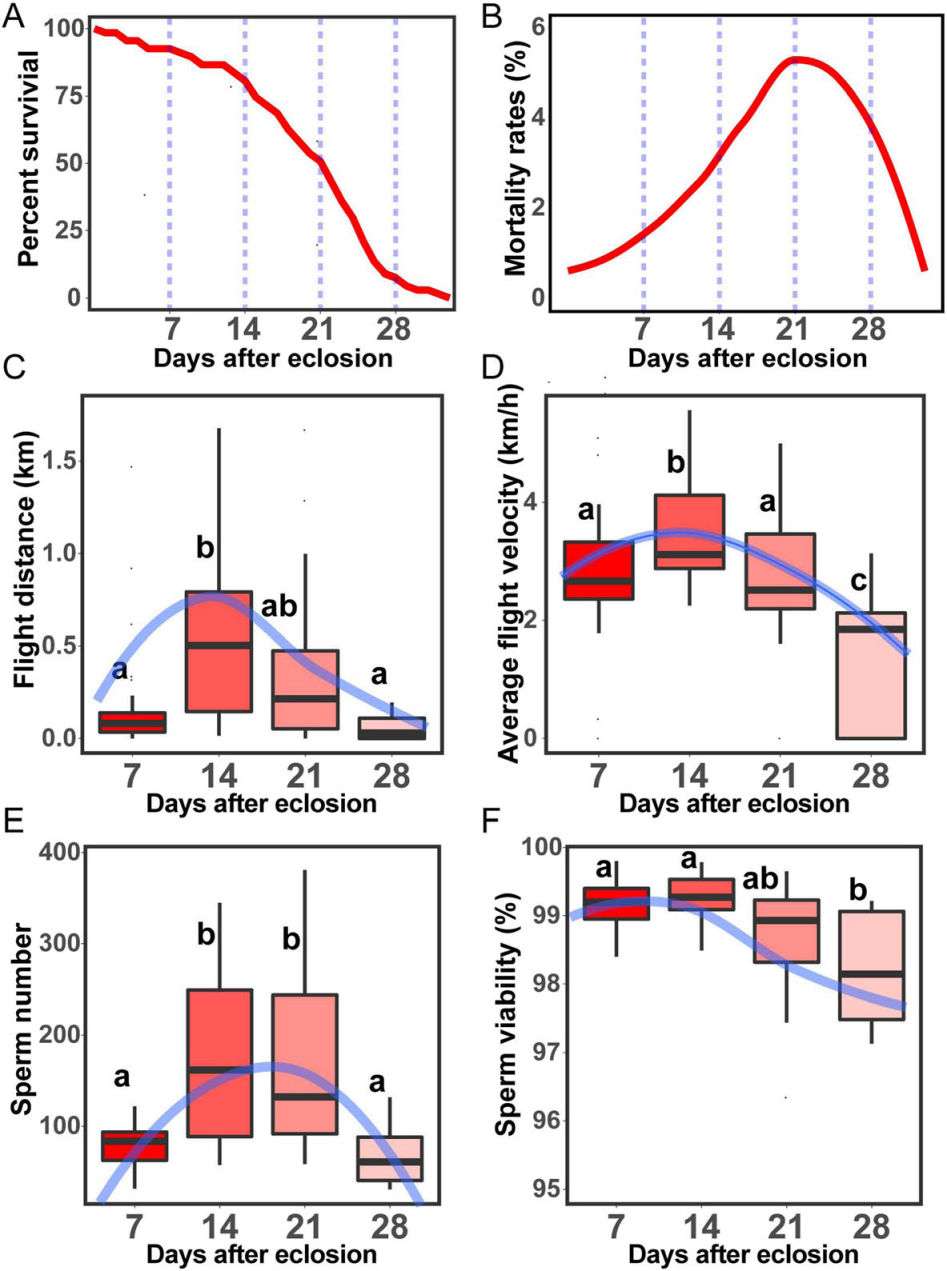
Aging-dependent physiological phenotypes in adult male locusts. **a** Survival curve of adult male locusts. The blue dashed lines divide the study period at a 7-day interval. *n* = 67. **b** Loess curve fitting of the mortality curve of adult males. **c**, **d** Flight performance of adult males at different ages. Flight distance (**c**) and average flight velocity (**d**) of adult males at 7, 14, 21, and 28 D. Significant differences are denoted by letters (one-way ANOVA, *P* < 0.05). The center line of the boxplots represents the median value, and the bounds of the box represent the 75th and the 25th percentiles. Blue lines represent the loess curve fitting of flight performance with age. **e**, **f** Sperm state of adult males at different ages. Stored sperm number (**e**) and sperm viability (**f**) of adult males at 7, 14, 21, and 28 D. Significant differences are denoted by letters (one-way ANOVA, *P* < 0.05)

The lifespan of adults is the most important parameter in aging. The median and maximum adult lifespans of male locusts were 22 and 33 days, respectively (Fig. 1a and Table S1). The mortality rate accelerated from eclosion to approximately 21 D (D = days after eclosion) with a subsequent deceleration (Fig. 1b).

Flight ability was assessed at 7, 14, 21, and 28 D. Four parameters, namely, flight distance (FD) in 1 h, flight duration in 1 h, average flight velocity (AV), and maximum flight velocity, were recorded to assess the flight ability. All the four parameters peaked at 14 D, steeply declined until 28 D, and declined in FD and AV from 14 D to 28 D by 92.1 and 59.1%, respectively. (Fig. 1c and d; Figs. S1A and S1B).

The effect of age on the number and viability of stored sperm were observed to assess sperm quality. The number of stored sperm peaked at 14 D, remained stable until 21 D, and steeply declined at 28 D; a decline of 62.4% was observed between 14 and 28 D (Fig. 1e). The proportion of viable sperm progressively declined from 14 D, but the decline was minimal, with only 1.1% between 14 and 28 D (Fig. 1f).

These results indicated that the key life history traits, flight ability and sperm state displayed clear aging-related declines in male adult locusts.

### Similarity of aging-related genes between locusts and canonical model species

**Fig. 2:**
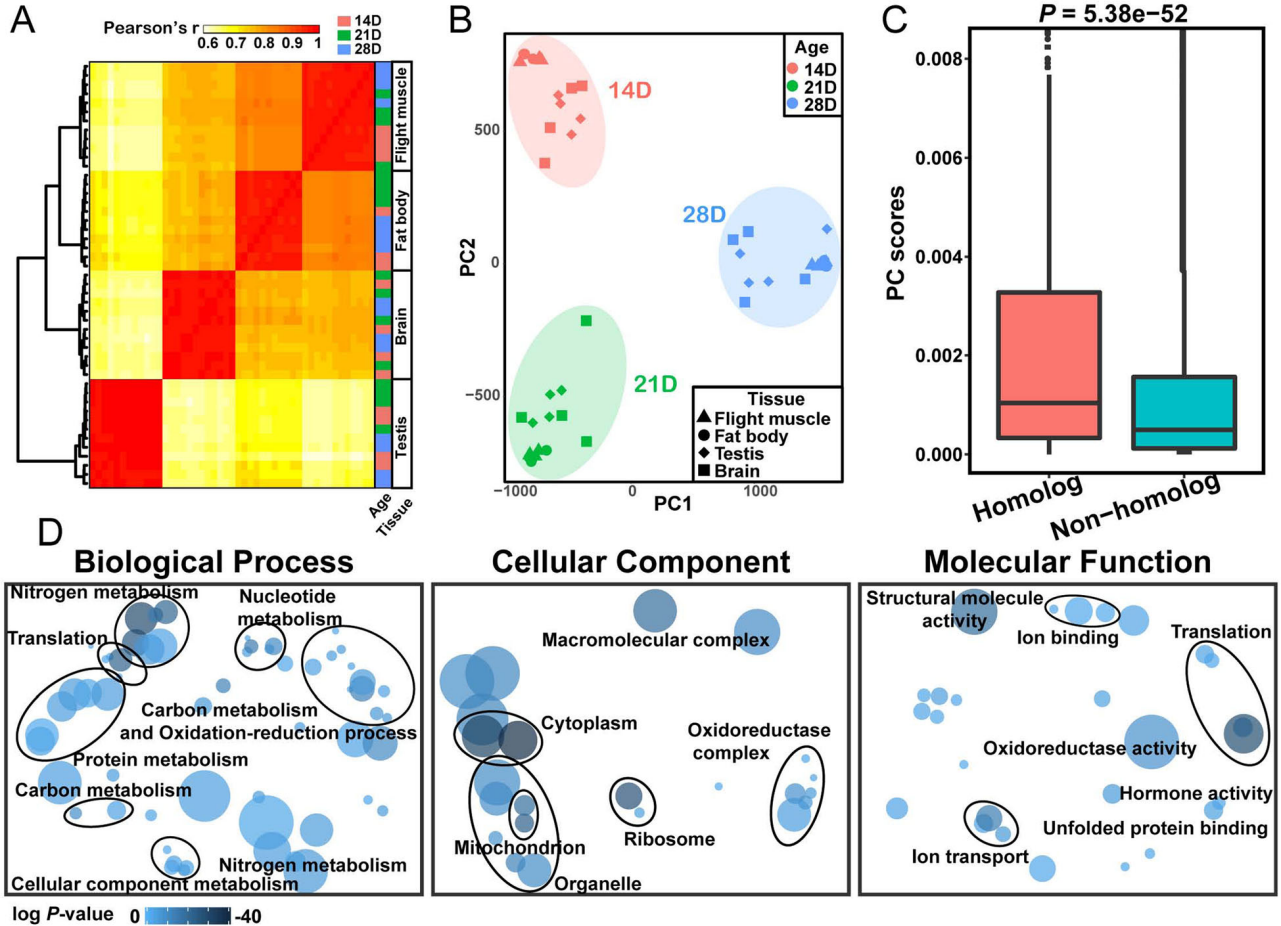
Similarity of aging-related genes between locusts and canonical model species. **a** Gene expression heatmap. Cell colors represent the Pearson’s correlation coefficient of gene expression values from two samples. A deep red color indicates high positive correlation. The red, green, and blue squares at the right panel denote the three time points. **b** Samples classified using the adjustment for confounding principal component analysis (AC-PCA) depending on the age time point. Dots represent the samples shaped on the basis of organs and colored in accordance with ages. **c** Boxplot shows the absolute PC2 scores of locust genes with or without identified orthologs in the GenAge database. The PC2 scores are from AC-PCA. The *P*-values determined by Mann–Whitney U test was denoted. **d** Gene Ontology (GO) enrichment analysis based on top 2000 genes ranked on the basis of the absolute PC2 values of AC-PCA. The shading of circles indicates the relative significance of the GO term (a darker shade indicates a more significant GO term), and the size of the circles corresponds to the number of genes annotated to the term (a larger size indicates a higher number)

Spatiotemporal gene expression profiles were determined using RNA-seq to elucidate the aging molecular signatures of locusts. As the male locusts at 14 D performed best in flight and sperm state, 14-D old locusts were chosen as a mature adult reference for the subsequent analyses. A total of 47 RNA-seq libraries were constructed from the flight muscle, fat body, testis, and brain at 14, 21, and 28 D. High correlations (> 0.95) among the samples from the same organs indicated the reliable quality of the RNA-seq datasets (Fig. 2a).

The degree of similarity of aging-related genes between locusts and other model species was assessed to evaluate the extent of the conservation of aging mechanisms between them. A total of 1426 aging-related homology genes in locusts were identified by comparing the locust gene sets with the GenAge database. Then, AC-PCA analysis based on transcriptomes was performed to assess the locust genes in accordance with their contribution to aging; the higher the score achieved in this analysis, the closer the relationship with aging. The second principal component (PC2) can discriminate three age points: 14 D, 21 D, and 28 D (Fig. 2b). Thus, the PC2 scores were used to represent the differences in gene expression among the age points. The PC2 scores of these aging-related homology genes were remarkably higher than those of other genes in the locust genome, revealing that the aging genes of other species also showed a close relationship with locust aging (Mann–Whitney U test, *P* = 5.38e− 52; Fig. 2c). For each organ, the corresponding PC scores of the aging-related homology genes were higher than those of the other genes in the locust genome (Mann–Whitney U test, *P* < 7e− 52; Fig. S2). GO enrichment analysis of the top 2000 genes with the highest absolute PC2 scores showed a clear enrichment for canonical aging-related pathways, including carbon and nitrogen metabolism, oxidation-reduction process, mitochondrion, ribosome, structural molecule activity, and ion homeostasis (Fig. 2d). Collectively, the aging-related genes displayed transcriptomic similarity between the locust species and other model species.

### Organ-specific patterns of aging-related transcriptomic profiles in locusts

**Fig. 3:**
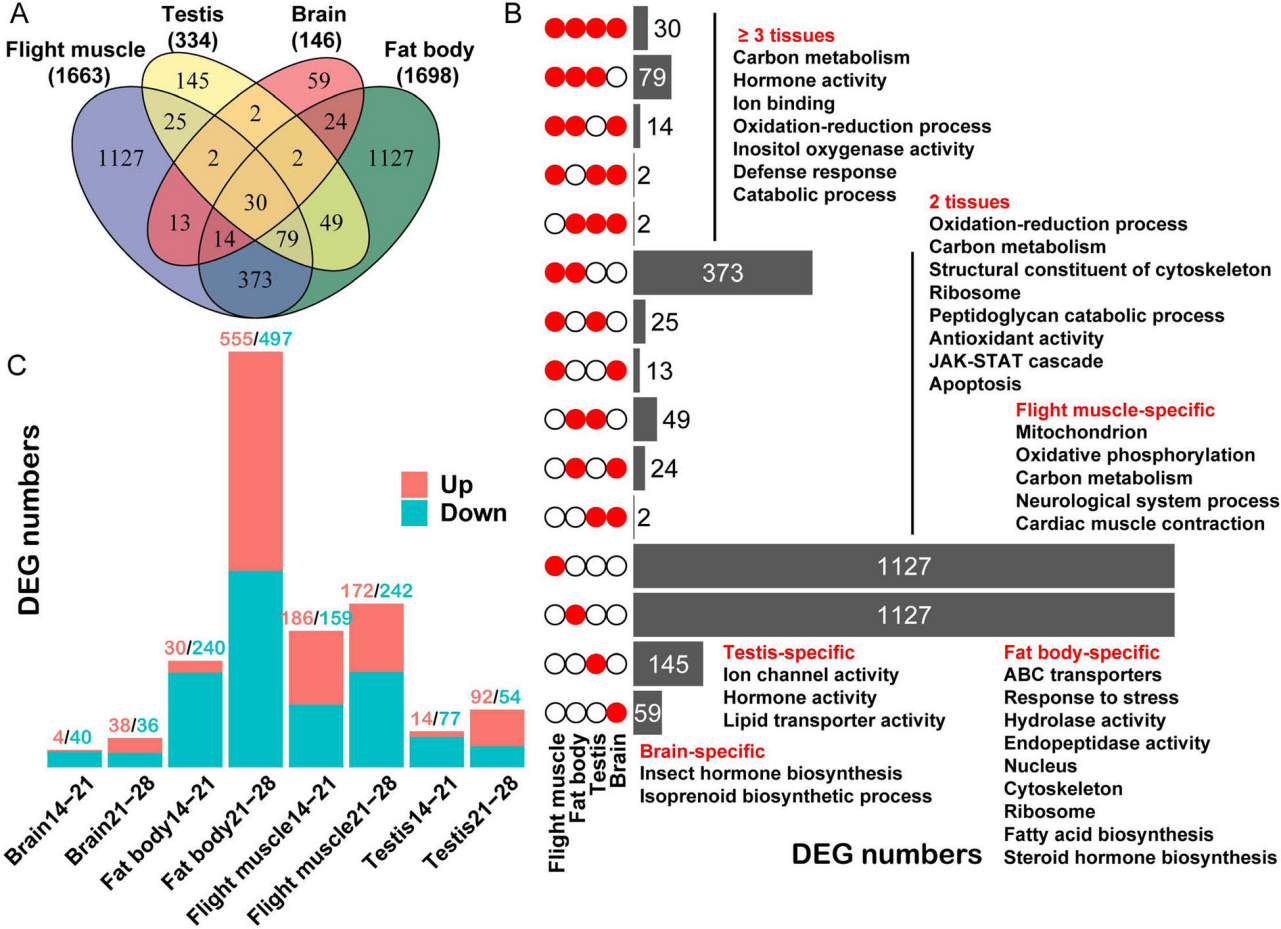
Temporal-spatial patterns of aging-related differentially expressed genes (DEGs). **a** Venn diagram of aging-related DEGs of different organs. The total number of organ-specific DEGs is shown in parentheses. **b** Bar chart illustrating the DEGs of different organs. The matrix of solid and empty circles at the left illustrates the “presence” (solid red) or “absence” (empty) of the organ in each intersection. The bar at the right represents the number of DEGs in each intersection. The representative enriched GO and Kyoto Encyclopedia of Genes and Genomes (KEGG) terms for the gene sets are shown on the right. **c** Bar chart illustrating the number of DEGs at 14–21 and 21–28 D within each organ. The DEGs are classified as “up” (marked in red) for the genes with an increased expression during this period and “down” (marked in blue) for the genes with a decreased expression in this period. The numbers at the top of the bar correspond to the numbers of DEGs

Gene that significantly changed expression at any two time points was defined as aging-related differentially expressed gene (DEG). The aging-related DEGs of four different organs, flight muscle, fat body, testis, and brain were compared to investigate the organ-level transcriptional profiles. A total of 3071 genes significantly altered their expression levels in at least one organ upon aging, including 1663 flight muscle DEGs, 1698 fat body DEGs, 334 testis DEGs, and 146 brain DEGs (Fig. 3a). Among these DEGs, only 4.14% (127) were commonly identified across three or four different organs, which were enriched for carbon metabolism, oxidation-reduction process, and defense response (Fig. 3b). In addition, 15.83% (486) of the total DEGs were shared by two organ types. Among 486 DEGs, 373 were shared by flight muscle and fat body. The DEGs shared by two organs were mainly enriched for metabolic process, cellular composition, and stress and defense responses (Fig. 3b). 80.04% of the total DEGs (2458) only existed in single organ (Fig. 3b). The flight muscle-specific DEGs were significantly enriched for mitochondrion (*P* = 2.79e− 19, GO enrichment). The fat body-specific DEGs were mainly enriched for the ATP-binding cassette (ABC) transporters (detoxification proteins) and stress responses. The testis- and brain-specific DEGs were involved in endocrine regulation (Fig. 3b). Moreover, declines in the expression of genes involved in mitochondrion and carbon metabolism were particularly evident in aged flight muscle, while increases in phagocytosis (i.e., endocytosis, phagosome, and lysosome) were evident in aged fat body (Fig. S3). The organ-specific DEGs were obviously correlated with the physiological roles specific to the corresponding organ.

The DEGs of each organ were classified into two groups depending on 14–21 and 21–28 D time periods to investigate the temporal changes in transcriptomics with aging (Fig. 3c). Among all the comparisons, the fat body at 21–28 D showed the largest expression change, which was mainly involved in the upregulation of ABC transporters, stress and defense responses, and the downregulation of lipid metabolism (Fig. 3c and S4A). The number of downregulated DEGs was relatively stable between the two time periods across the organs. Upregulated DEGs were mainly detected at 21–28 D in organs except flight muscle, which exhibited 186 genes upregulated at 14–21 D (Fig. 3c). These 186 upregulated genes were enriched for aromatic amino acid metabolism and defense response (Fig. S4B).

### Co-expression network analysis of aging-related transcriptomic profiles

**Fig. 4:**
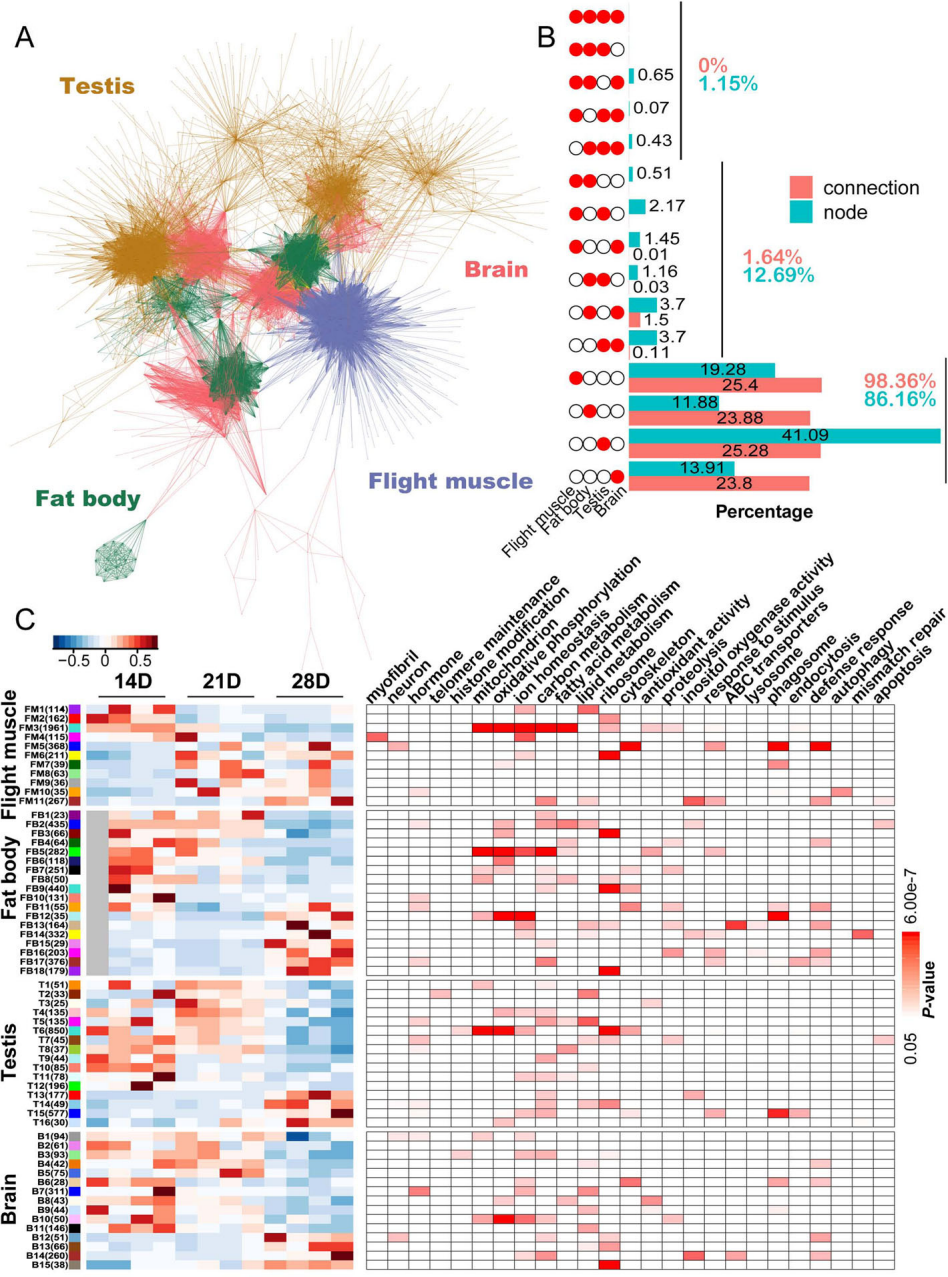
Co-expression network analysis of aging-related transcriptomic profiles. **a** Visual network constructed using the top 5000 connections of each network from four organs. Edge colors represent different organs. **b** Bar chart illustrating the numbers of connections and nodes across the four organs. The matrix of solid and empty circles at the left illustrates the “presence” (solid red) or “absence” (empty) of the organ in each intersection. The bar at the right represents the percentage of connections and nodes in each intersection. The total percentages of connections and nodes that are shared by at least three organs, two organs, and unique to a single organ are shown on the right. **c** Heatmap of eigengenes from selected aging-biased modules and corresponding enriched GO and KEGG terms in each organ. The left panel is the heatmap of module eigengenes associated with age status. The names and number of genes (shown in parentheses) of modules are listed on the left. The high and low eigengene values of each module are marked in red and blue, respectively. The right panel is the grid of enriched GO and KEGG terms in modules, and red boxes represent modules with significant enrichment (GO terms with *P* < 0.01 and KEGG terms with *P* < 0.05). The color density represents the corresponding *P-*value (a deeper red color indicates a more significant term)

Five co-expression networks, including a total network containing all organs and four dependent networks from each organ, were constructed using all aging-related genes to obtain a global view of the aging-related transcriptomic profiles across organs. The four organ-specific networks were merged into a visual network comprising 1380 nodes and 19,687 connections to assess the degree of expression similarity across organs (Fig. 4a). Most of these nodes (86.16%) were observed in one organ, with only 12.69% (175) and 1.15% (16) for two and three organs, respectively (Fig. 4b). The network connections also displayed similar organ specificity, with up to 98.36% (19,365) for one organ, 1.64% (322) for two organs, and none shared by three or four organs (Fig. 4b). Subsequently, the aging-biased modules from each organ network were identified, and some modules with similar patterns across all organs contained genes with similar functions. For example, the FM3 module of the flight muscle, the FB5 module of the fat body, the T6 module of the testis, and the B10 module of the brain, which were all downregulated during aging, were involved in mitochondrion and carbon metabolism (Fig. 4c). Other modules had organ-specific characteristics. For example, the FM4 and FM10 modules of the flight muscle were enriched for myofibril and autophagy, while the FB13 and FB14 modules of the fat body were enriched for ABC transporters and mismatch repair, respectively (Fig. 4c).

### Aging-dependent cellular phenotypes in locusts

**Fig. 5:**
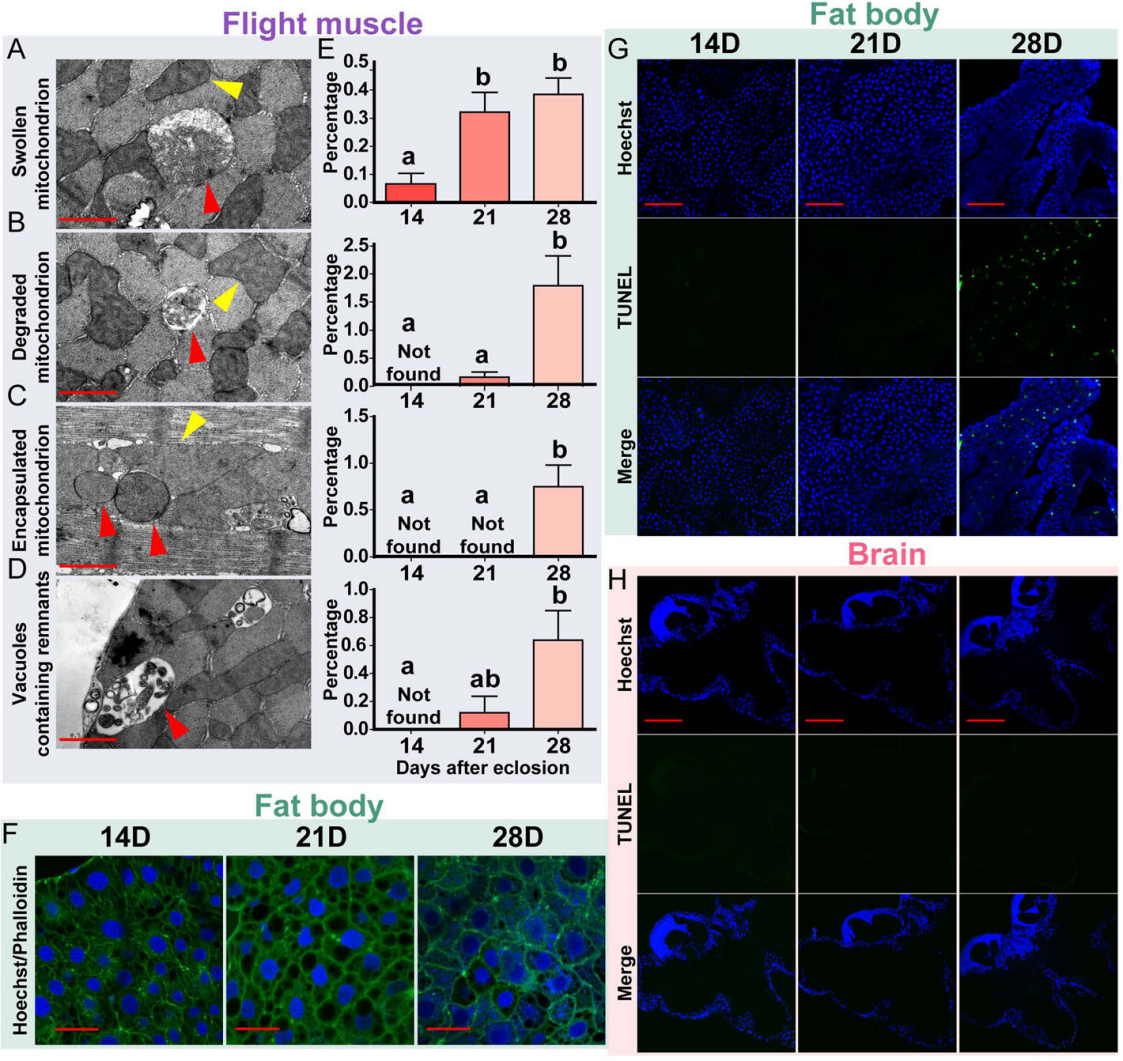
Aging-related cellular phenotypes at various organs. **a**–**d** Representative morphologies of abnormal mitochondria and vacuoles containing remnants in aged flight muscle. The yellow triangles indicate normal mitochondria, whereas the red triangles indicate abnormal mitochondria or vacuoles. Bar length = 1 μm. **e** Percentages of abnormal mitochondria and vacuoles containing remnants shown in (**a**–**d**) at 14, 21, and 28 D. The bar graphs with quantifications belong to the abnormity on the same line. The values are expressed as mean ± S.E.M. Significant differences are denoted by different letters (one-way ANOVA, *P* < 0.05). **f** Morphological characteristics of fat body cells at 14, 21, and 28 D. Blue, Hoechest 33,342 (cell nuclei); green, phalloidin (F-actin). Bar length = 50 μm. (G,H) Apoptotic cell detection in fat body (**g**) and brain (**h**) at 14, 21, and 28 D. Blue, Hoechest 33,342 (cell nuclei); green, TdT-mediated dUTP nick end labeling (TUNEL) (apoptotic cells). Bar length = 200 μm. The statistical analyses of abnormal nuclei shown in (**f**, **g**, **h**) are in Fig. S5

Transcriptome analyses revealed that flight muscle and fat body changed greatly with aging, but brain changed little. The predominant aging transcriptional signatures of flight muscle and fat body were dramatic change in expression of mitochondrion-related genes and phagocytosis and detoxification genes, respectively. To reveal the aging-related cellular changes, we further detected the aging-related mitochondrial states in flight muscle and cellular states in fat body. Transmission electron microscopy showed that the ratio of abnormal mitochondria in the flight muscle, including swelling (Fig. 5a), degradation (Fig. 5b), and encapsulation (Fig. 5c), increased with aging. At 14 D, only 0.07% of the mitochondria was swollen, and no degraded and encapsulated mitochondria were observed (Fig. 5e). However, the proportions of swollen mitochondria with disrupted cristae reached 0.32 and 0.38% at 21 and 28 D, respectively (Fig. 5e). The proportions of degraded and encapsulated mitochondria intensively increased at 28 D, reaching 1.79 and 0.75%, respectively (Fig. 5e). In addition, the number of vacuoles containing remnants increased with aging, reaching 0.64% at 28 D (standardized by the number of mitochondria, Fig. 5d and e). For the fat body cells of 28-D old locusts, the boundaries and round shapes of the cell nuclei changed, and DNA diffused in the cellular matrix (Fig. 5f and Fig. S5). Apoptosis also considerably increased at 28 D (Fig. 5g and Fig. S5). Corresponding to the little transcriptional change in brain aging, aged brains showed no increase in apoptosis (Fig. 5h and Fig. S5). These results suggested significant cellular alterations in the aged flight muscle and fat body but not in the brain.

### Functional assessments of aging-related genes in locusts

**Fig. 6:**
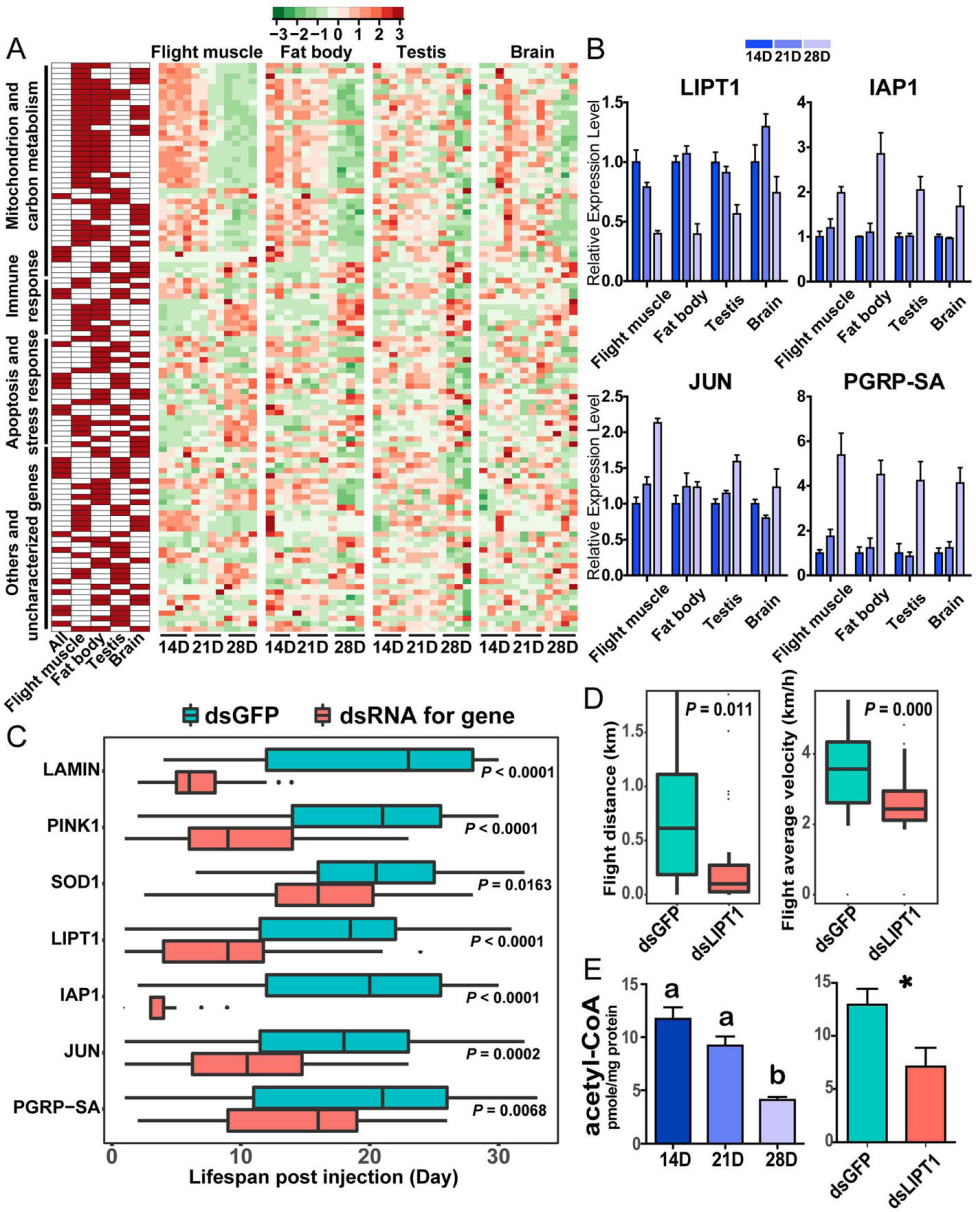
Functional assessments of aging-related genes in locusts. **a** Expression patterns of hub genes common to at least two networks. The heatmap on the left illustrates whether the gene is the hub gene of the corresponding network. The presence and absence are denoted by red and empty, respectively. The functional categories of genes are shown on the left. Four heatmaps on the right show the relative expression levels of the corresponding genes upon aging in the four organs. The high and low expression levels of each gene are marked in red and green, respectively. **b** Relative mRNA expression levels of four representative genes during aging across the four organs based on transcriptome data. **c** Lifespan effects of the knockdown of the selected genes. Blue and red boxplots refer to the lifespan of locusts treated with dsGFP or dsRNAs for the selected genes, respectively. The center line of the boxplots represents median value, and the bounds of the box represent the 75th and the 25th percentiles. The *P*-values examined via the Mantel–Cox tests are annotated on the right. **d** Flight performance assessment after *LIPT1* knockdown. The *P*-values evaluated via Student’s *t* test are annotated. **e** The level of acetyl-CoA in flight muscle during aging and after *LIPT1* knockdown. Significant differences are revealed using one-way ANOVA, *P* < 0.05 (left) and Student’s *t* test, *P < 0.05 (right)

108 aging-related genes were screened in accordance with the criteria satisfied by the hub genes in at least two co-expression networks and the top 1000 genes in the corresponding AC-PCA (Fig. 6a and Supplementary file 8). Among them, four representative genes, namely, lipoyltransferase 1 (*LIPT1*), death-associated inhibitor of apoptosis 1 (*IAP1*), transcription factor *JUN*, and peptidoglycan recognition protein SA (*PGRP-SA*) with significant expression changes during aging, were selected for functional studies (Fig. 6b and S6). Three well-known aging genes, namely, *LAMIN*, PTEN-induced putative kinase 1 (*PINK1*), and *SOD1*, which were aging-related DEGs in at least one organ (Fig. S7), were also selected for functional studies. After dsRNA injection, the expression levels of these seven genes were dramatically reduced in all four organs of locusts (Fig. S8). The knockdown of all seven genes significantly shortened the locust lifespan. The median lifespans decreased by 73.9, 57.1, 22.0, 51.4, 80.0, 41.7, and 23.8% for *LAMIN*, *PINK1*, *SOD1*, *LIPT1*, *IAP1*, *JUN*, and *PGRP-SA*, respectively (Fig. 6c and Table S1). After *LIPT1* knockdown, the flight parameters FD and AV of the 14 D-old adult males declined by 63.0 and 29.3%, respectively (Fig. 6d). In addition, the flight muscles of old locusts and *LIPT1*-knockdown locusts both displayed decreased level of acetyl coenzyme (CoA), a key intermediate involved in different metabolic pathways (Fig. 6e). Therefore, all these selected aging-related genes can affect lifespan, and *LIPT1* can affect flight ability and metabolism in locusts.

## Discussion

Here, the organ-level aging signatures of the migratory locust with physiological, cellular, and transcriptional changes were sketched. The aging-related traits of locusts were highly similar to several model species [27, 28]. However, some distinct aging transcriptional features were also characterized, including increased autophagy in flight muscle, activation of detoxification in fat body, and little transcriptional and physiological changes in brain.

The results showed that locusts have largely conserved aging characteristics with other species. Similar to other animals [14, 28–30], locust aging was associated with significant declines in locomotor performance, sperm state, and mitochondrial functions, and increase in apoptosis. A significant similarity in aging-related genes and pathways was identified between locusts and other species. The aging characteristics of insects vary considerably depending on species. For example, *Drosophila* displayed aging-related functional declines, such as decreased flight ability and reduced learning and memory ability [29], whereas old honey bee workers showed no performance decline in locomotion, learning or responsiveness to light or sucrose, and the old minor workers of ant *Pheidole dentata* showed constancy in behavioral performance and the absence of significant aging-related neural declines [31, 32]. The present study suggested that multiple typical aging characteristics were retained in locusts.

The organ-specific aging vulnerability of locusts was mainly characterized by significant cellular and transcriptional alterations in the aged flight muscle and fat body. The flight muscle and fat body represent the organs closely involved in energy metabolism and stress responses [33]. The aging-related transcriptional changes in flight muscle and fat body of locusts were prominently associated with mitochondrion, carbon metabolism, and detoxification. Thus, the organ-specific aging trajectories in locusts may reflect their intense homeostatic disruptions of metabolism and stress responses in advanced age. However, several model species, such as rat and *Drosophila*, have been reported to display different organ-specific aging trajectories. The transcriptional profiles of rat testis were particularly sensitive to aging compared with the muscle and brain [34], while the aged fat body of *Drosophila* displayed less transcriptional change than the brain and testis [21]. In addition, there are still some genes showed aging-related expression changes across organs, which may represent the conserved markers of cellular senescence, such as OXPHOS and defense response [35]. Noteworthy, most shared DEGs of two organs are from flight muscle and fat body, which contribute to most enriched terms. Therefore, the result of enrichment analysis of DEGs shared by two organs may have limited ability to reflect functional changes across all organs.

For the aging of flight muscle in locusts, the predominant characteristics were the occurrence of mitochondrial abnormalities, a dramatic decline in genes involved in mitochondrion and carbon metabolism, and the increase in genes associated with autophagy. The flight muscle of *Drosophila* also has specific vulnerability to aging, and it is characterized by marked transcriptional changes, damage accumulation, and functional decline [21, 29, 36]. Given that insect flight muscles have the highest respiratory activity among all animal organs [37], the specific aging susceptibility of flight muscles may be due to the rapid accumulation of oxidative damage associated with hyperactive respiratory metabolism [38]. Increasing evidence supporting the aging effect of intensifying oxidative stress has been shown by paraquat treatment that could lead to some phenotypes similar to aged flight muscle, including mitochondrial swelling and downregulation of mitochondrial genes [39, 40]. However, unlike some model species [41], the aged flight muscle of locusts probably has increased degree of autophagy to protect against aging-related damage accumulation.

The aged fat body in locusts was accompanied with intense transcriptional activation of ABC transports and phagocytosis and marked increase in cell nucleus abnormalities and apoptosis. In the aged fat body of *Drosophila*, a similar increase in cell nucleus abnormalities and apoptosis occurred [36, 42]. Because phagocytosis is required for the clearance of apoptotic bodies and cell debris [43], the upregulation of genes involved in phagocytosis may be in response to the increment of apoptotic corpse in the fat body of aged locusts [44]. The upregulation of ABC transporters with ages is unique to locust fat body. In insects, ABC transporters belong to a superfamily of proteins involved in the transport of specific molecules and detoxification exporting the conjugated toxins out of cells [33]. A previous work has proven that the expression of ABC transporters in insect fat body increased after toxin exposure [33]. Thus, the upregulation of ABC transporters in the aged fat body of locusts implicated the possible accumulation of toxic compounds in old locusts.

The transcriptional profile in the locust brain only slightly changed during aging. Only 146 aging-related DEGs were found in the locust brain, much less than the DEG numbers in other organs. Unlike locusts, *Drosophila* brain displayed a greater aging-related expressional change than testis and fat body, and rat brain had more aging-related DEGs than liver [21, 45]. In addition, increased sign of apoptosis with aging was not detected in the locust brain, which was different from the observation in mammals [46]. Therefore, locust brains may possess particular antiaging characteristics. Indeed, the central nervous system of locusts have some unique features, such as very large number of microglomeruli in the antennal lobes [47]. Several studies on *Drosophila* and humans have suggested that aging mechanisms in brains may be specific to cell type; for example, glial cells, not neuronal cells, are sensitive to aging at the transcriptional level [48, 49]. Thus, the species-specific traits of cell types, such as low proportion of glial cells in locusts [50], may be the partial reason for the little change in transcriptomic profile upon aging in the locust brain. Certainly, the possibility of aging-related changes at other levels in locust brains cannot be excluded, such as post-transcription, the structure of axon terminations, or learning and memory behaviors, which deteriorate over the lifespan in *Drosophila* [29, 51].

By using AC-PCA and WGCNA methods, three genes involved in stress and immune responses (i.e., *JUN*, *IAP1*, and *PGRP-SA*) and one metabolism-related gene (i.e., *LIPT1*) were screened to show extraordinary closely relationships with locust aging. *JUN* and *IAP1* have been reported to play important roles in apoptosis, inflammation, and metastasis [52, 53]. Their expression levels are induced by various stress stimulations and closely related with many human cancers [53–55]. However, the effects of *JUN* and *IAP1* on aging have not been confirmed. The upregulation of *JUN* and *IAP1* in all four organs of old locusts implicated that locust aging may be accompanied by systemic stress signals. *PGRP-SA* is an activator of Toll signaling, wherein the roles in aging and lifespan have not been studied in depth [56]. The aging-dependent upregulation of *PGRP-SA* indicated the activation of the Toll signaling pathway in old locusts, which may be attributed to increased risk of bacterial infection caused by gregarious living condition and immunosenescence [57]. The lifespan shortening after knockdown of *JUN*, *IAP1*, and *PGRP-SA* indicated the indispensable roles of stress responses and immune activation in the survival of old locusts. Knock down of *LIPT1* not only induced lifespan shortening, but also decreased flight ability and acetyl-CoA level in locusts. *LIPT1* catalyzed lipoylation of mitochondrial protein and further regulate mitochondrial metabolism [58]. In humans, *LIPT1* deficiency reduced lipoylation of pyruvate dehydrogenase (PDH) and the level of PDH product acetyl-CoA, and induced metabolic abnormalities [59]. The decrease in *LIPT1* expression in old locusts and reduced flight ability and acetyl-CoA level after *LIPT1* knockdown suggested the involvement of *LIPT1* in locomotor activity and metabolism, possibly through aging-dependent decline of mitochondrial lipoylation. Noteworthy, our results revealed that regardless of gene expression with aging, gene knockdown all induced lifespan reduction. The shortened lifespan after genetic manipulations may not reflect accelerated aging, but only means increased mortality in absence of functional genes. Whether these genes are really involved in the regulation of aging needs further studies.

Here, several methods, including AC-PCA, DEG analysis, WGCNA, and enrichment analyses, were conducted to reveal the aging-related expression characteristics in locusts. Multi-method strategy of transcriptome analyses is to avoid the drawbacks and limitations of a single method, improve confidence in results and yield a more comprehensive understanding of our results. Filtering input genes by differential expression in WGCNA may lead to the creation of a few highly correlated modules and failure to construct networks correctly (https://horvath.genetics.ucla.edu/html/CoexpressionNetwork/Rpackages/WGCNA/faq.html). However, WGCNA here was conducted to reveal the transcriptional pattern of aging-related genes obtained from multiple data source including AC-PCA and DEG analysis. Therefore, 4852 aging-related genes including DEGs of all organs and high-scoring genes from different AC-PCA analyses, instead of all genes were used in network construction. Furthermore, in our results, the WGCNA results were also supported by DEG analysis.

Several aging characteristics of gregarious locusts may be attributed to the crowding stress. The study on desert locusts proved that the lifespan of gregarious locusts is 44% shorter than solitary ones, suggesting that locusts age fast or unhealthy under crowding [12]. Our study revealed dramatic increase in mitochondrial abnormalities and change in gene expression in flight muscle during gregarious locust aging. Crowding may contribute to these phenotypes by enhancing behavioral activity and generation of reactive oxygen species [5, 38, 40, 60]. Further researches are needed to clarify the aging differences between gregarious and solitary locusts, which serves as an ideal model to study the effects of crowding on aging.

## Conclusion

The aging features of the migratory locust from physiological to transcriptional levels were characterized in the present study. Locust aging was accompanied by remarkable impairments in flight ability and sperm state. Although the aging-related genes of locust were similar with those of canonical model species, several organ-specific aging features, such as intensive expression changes in flight muscle and fat body and little transcriptional changes in brain, were unique to locusts. The expression of genes related to mitochondrion changed greatly in flight muscle, and the expression of genes related to detoxification and phagocytosis changed greatly in fat body. Cellular assessments revealed increase in mitochondrial and nuclear abnormalities in aged flight muscle and fat body, but not in brain. In addition, the roles of four aging-related genes (i.e., *JUN*, *IAP1*, *PGRP-SA*, and *LIPT1*) involved in apoptosis, immunity, and mitochondrial dysfunctions in affecting locust lifespan, locomotion and metabolism are highlighted. In summary, locusts represent a promising study model for aging biology with remarkable aging features, which could expand the understanding of the molecular basis of aging-related changes in metabolism and stress responses.

## Methods

### Insect rearing

All the insects used in the experiments were reared in the same locust colonies at the Institute of Zoology, Chinese Academy of Sciences, Beijing, China. The locusts were reared in the gregarious phase under a 14:10 light/dark photo regime at 30 ± 2 °C as previously described [61]. The diet included a continuous supply of dry wheat bran with fresh wheat seedlings provided once per day.

### Lifespan assay

The locusts were kept as described above for all lifespan assays. For the lifespan assessment of the untreated locusts, colonies were inspected daily, and the selected newly eclosed males were marked on the pronotum. After eclosion, dead male locusts were removed and recorded daily. A total of 67 replicates were chosen for lifespan measurement. For lifespan assessment after RNAi, at least 58 adult males 2 days after eclosion were selected for each gene. The locusts were injected with double-strand RNA (dsRNA) by an interval of 4 days until death. The survival rate was calculated by determining the percentage of surviving locusts. The *P-*value for comparing the survival curves between two groups was determined using a log-rank Mantel–Cox test. GraphPad Prism was used to perform statistical analyses. The full details of the lifespan trials are presented in Table S1.

### Flight performance

The flight performance of adult male locusts was measured using a custom-built computerized flight mill system. The length of the radial horizontal beam was 12 cm. The locusts were tethered to the end of the rotor. The interruption of an infrared beam by the rotating arm generated an electrical signal that was recorded by a computer. A warm air stimulus was applied when the electrical signal stopped for 1 min. The assay time for a single locust was 1 h. Flight distance, flight duration, average flight velocity (flight distance/flight duration), and maximum flight velocity were recorded. The flight mill setup was maintained at 30 ± 2 °C. Flight measurements were conducted between 14:00 and 17:00. 20–38 locusts were examined for each age group, and 76 locusts were examined after RNAi manipulation. Differences were evaluated by one-way ANOVA followed by Tukey’s test for multiple comparisons or by *t* test with SPSS 17.0.

### Sperm count and viability

A single seminal vesicle (the male’s sperm store) was dissected and immediately placed in Grace’s insect buffer (Thermo Fisher, Massachusetts, USA). Fat body was carefully removed from the seminal vesicles. Then, we ruptured the seminal vesicles in 100 μL of fresh Grace’s medium. All sperm were collected and digested with 40 μL of trypsin (15,050,057, Gibco, Grand Island, NY, USA) for 2 min. Digestion was then stopped with 40 μL of fetal bovine serum (FSS500, ExCell Biology, Carlsbad, California, USA). The samples were centrifuged at 1200×g for 25 s to pellet the sperm. The supernatant was removed, and the pellet was resuspended in 50, 100, or 200 μL of medium depending on the estimated concentration of semen. Three tubes (25 μL each) of diluted semen were stained with 0.2 μL of SYBR-14 dye (1:25 in medium) for 10 min and 2.5 μL of propidium iodide (live/dead sperm viability kit, L-7011, Molecular Probes, Oregon, USA) for 7 min at 28 °C. After the specimens were stained, 10 μL of the sample was placed on a clean slide and covered with 10 mm round cover slips. Ten images of each sample were examined under a fluorescence microscope (DFC425C, Leica Microsystems, Wetzlar, Germany). The sperm count of each individual image was quantified using ImageJ. The sperm viability was calculated by determining the percentage of live sperm. Differences were evaluated by one-way ANOVA followed by Tukey’s test for multiple comparisons. About 8–17 individuals were performed for each age group.

### RNA-seq and data processing

Four types of organs (i.e., brain, fat body, testis, and flight muscle) of adult male locusts were collected at 14, 21, and 28 days after eclosion. Three or four biological independent replicates were performed for each group. Each biologically independent replicate was a pool of organs obtained from four individuals. Total RNA was extracted by using TRIzol reagent (15,596,018, Invitrogen, Carlsbad, USA). cDNA libraries were prepared in accordance with the protocols of NEBNext® Ultra™ RNA Library Prep Kit for Illumina®, and sequenced on Illumina Hiseq 2000. Raw reads with low quality and adaptor sequences were filtered using Trimmomatic with default parameters [62], and the clean reads were mapped to the genome reference of locusts [26] by using TopHat2 [63]. The number of unique mapped reads to every gene model was counted using HTseq [64]. DEGs were detected using edgeR package [65], defined as fold change > 2, and adjusted with *P* < 0.1. The gene expression level was measured as reads per kilobase per million reads (RPKM). RPKM values of all genes were listed in Supplementary file 2. RNA-seq data were deposited at NCBI SRA database with BioProject ID PRJNA562411.

### The adjustment for confounding principal component analysis (AC-PCA) and characterization of aging-related homology gene

AC-PCA analysis was performed using the acPCA package [66] as previously described [67]. The *acPCA* function was run with a linear kernel, and the input Lambda parameter was tuned using the *acPCAtuneLambda* function. PC scores of all genes were listed in Supplementary file 3. A list of aging genes in model species, including human, mouse, fruit fly, worm, and yeast, was downloaded from the GenAge database [68]. The locust orthologs of these genes were determined by using inparanoid [69]. Aging-related homology genes of locusts were listed in Supplementary file 4.

### Co-expression network analysis

The genes whose expression varied among different time points in the four organs were selected to construct co-expression networks. These genes included 1) the DEGs among pairs of time points from the same organ and 2) the top 2000 genes ranked by the absolute scores of the selected PC from AC-PCA. The weighted gene co-expression network analysis (WGCNA) R package [70] was used to construct the co-expression networks. A signed adjacency matrix was calculated. For each pair of genes, a topological overlap was calculated on the basis of the adjacency matrix and used to measure the correlation between them. For each gene, connectivity was defined as the sum of connection strengths with other genes in the network. The highly interconnected genes were clustered as modules and represented by specific colors. A module eigengene was defined as the first principal component of a given module. It could be considered a representative of the gene expression profiles in a module. For each network, the 10% genes with the highest connectivity were defined as hub genes. The merged visual network was constructed using top 5000 connections of each individual networks from the four organs. The membership between genes and modules were listed in Supplementary file 5. The eigengene of each module were listed in Supplementary file 6. The hub genes of each network were listed in Supplementary file 7.

### Enrichment analysis

Enrichment analysis of the Gene Ontology (GO) and Kyoto Encyclopedia of Genes and Genomes (KEGG) for the supplied gene list was carried out based on an algorithm presented by GOstat [71, 72], and the whole annotated gene set in the locust genome was set as the background. *P*-value of the enrichment score was determined using the chi-square test. Fisher’s exact test was performed when any expected value of count was below 5. For GO enrichment analyses, the ancestor item was deleted from the results if one item was the ancestor of another and the lists of the enriched genes of these two items were the same. The Benjamini–Hochberg method was used to adjust for multiple testing [73] for each class. Only GO terms with *P* < 0.01 and KEGG terms with *P* < 0.05 were considered. The program REVIGO [74] was used to visualize the GO enrichment results of top 2000 genes with the highest absolute PC2 scores in AC-PCA analysis.

### Transmission electron microscopy

For every time point, three insects were used for TEM analyses. For every insect, at least two different regions of flight muscle were observed. Indirect flight muscles were dissected quickly, cut into small pieces, and fixed using 2.5% glutaraldehyde and 1% paraformaldehyde in 0.1 M PB buffer (pH 7.4) at 4 °C overnight. The tissues were postfixed in 1% OsO4 at 4 °C for 2 h, serially dehydrated in 30, 50, 70, 85, 95, and 100% acetone (four times) for 10 min in each procedure. Each sample was then infiltrated in 3:1 acetone: SPI-Pon 812 resin (Spi Supplies, West Chester, PA, USA) for 1 h, 1:1 acetone: SPI-Pon 812 resin for 1.5 h, 1:3 acetone: SPI-Pon 812 resin for 3 h, and 100% SPI-Pon 812 resin overnight. The samples were subsequently polymerized in 100% SPI-Pon 812 resin with DMP-30 at 60 °C for 48 h. Ultrathin sections with a thickness of approximately 70 nm were made using an ultramicrotome with a diamond knife and stained with uranyl acetate and lead citrate. Analysis was performed by using a JEM-1400 transmission electron microscope (JEOL, Tokyo, Japan). The number of mitochondria and vacuoles was quantified using ImageJ. The mitochondria and vacuoles observed by the same insect were added for statistical analysis. In sum, for 14 D locusts, 756, 290, and 1529 mitochondria were counted for each insect; for 21 D locusts, 950, 1590, and 560 mitochondria were counted, respectively; for 28 D locusts, 671, 456, and 374 mitochondria were counted, respectively. Differences were evaluated through one-way ANOVA followed by Tukey’s test for multiple comparisons. Three independent replicates were performed for each age group. Multiple comparison tests were conducted from *N* = 3.

### Fat body imaging and confocal microscopy

Fresh fat body was immediately fixed in PBS containing 4% paraformaldehyde overnight at 4 °C, permeabilized in PBS containing 0.3% Triton X-100 at room temperature for 30 min. Then, samples were incubated with 0.165 μM Alexa Fluor-488 phalloidin (A12379, Molecular Probes, Eugene, OR, USA) in 1% BSA-PBS in the dark for 30 min to stain F-actin. Cell nuclei were stained with 5 μM Hoechst 33342 (H3570, Molecular Probes, Eugene, OR, USA) for 10 min. The samples were imaged using a ZEISS LSM 710 confocal microscope (Zeiss, Oberkochen, Germany). At least three independent replicates were performed for each age group.

### TdT-mediated dUTP nick end labeling (TUNEL) assay

Fresh fat body was fixed in PBS containing 4% paraformaldehyde at 4 °C overnight and treated with proteinase K (10 μg/ml) at 37 °C for 40 min. The sections of the brains (15 μm) were cut with a cryostat (CM1950, Leica Microsystems, Wetzlar, Germany Germany), fixed with PBS containing 4% paraformaldehyde for 20 min, and washed with PBS at room temperature for 30 min. The fixed brains were permeabilized with 0.1% TritonX-100 at room temperature for 10 min. The fat body and the brain were stained by using an in situ cell death detection kit (11,684,817,910, Roche Applied Science, Indianapolis, IN, USA) in accordance with the manufacturer’s protocol. Nuclei were stained with 5 μM Hoechst 33342 (H3570, Molecular Probes, Eugene, OR, USA). Images were captured with a ZEISS LSM 710 confocal microscope (Zeiss, Oberkochen, Germany). TUNEL assays were performed with at least three independent replicates.

### RNAi assay

The double-strand RNAs of seven targeted genes were prepared by using a T7 RiboMAX Express RNAi system (Promega, P1700, USA) in accordance with the manufacturer’s instructions. dsRNA concentrations were determined with an ND-1000 spectrophotometer (NanoDrop, Wilmington, DE, USA), and dsRNA quality was verified through 1% agarose gel electrophoresis. For the lifespan assay, adult locusts were injected with 4 μg of 2 μg/μl dsRNA in the second ventral segment of the abdomen by an interval of 4 days until death. The dsRNA of green fluorescent protein (*GFP*) was used as the control in these assays. For flight performance assay and acetyl-CoA measurement after *LIPT1* knockdown, adult males at 11 D were injected with 4 μg of 2 μg/μl dsRNA in the second ventral segment of the abdomen, and were assessed at 14 D. The effects of RNAi on relative mRNA expression levels were investigated through quantitative polymerase chain reaction (qPCR) after injection for 72 h. The primers of the targeted genes for dsRNA synthesis are listed in Table S2.

### Quantitative PCR

Total RNA was prepared as mentioned above. RNA concentration was determined using an ND-1000 spectrophotometer (NanoDrop, Wilmington, DE, USA). RNA integrity was confirmed through 1% agarose gel electrophoresis. Moloney murine leukemia virus reverse transcriptase (Promega, M1701, USA) was used to prepare oligo (dT)-primed cDNA from 2 μg of total RNA. The relative expression of mRNA was quantified with SYBR green 1 Master Mix (04707516001, Roche Applied Science, Indianapolis, IN, USA) and LightCycler 480 (Roche, Mannheim, Germany). Amplification specificity was confirmed through melting curve analysis. Gene expression levels were normalized to gene expression of RP49, ACTIN, and HSP70. Expression data were analyzed using 2^−ΔΔC*t*^. Differences were statistically evaluated via a *t* test in SPSS 17.0. Quantitative reverse transcription PCR was performed in three to four biological replicates. Four individual samples were used in each biological replicate. The qRCR primers are listed in Table S2.

### Acetyl-CoA measurement

Fresh flight muscle (20–40 mg) of 3–4 individuals were dissected out and homogenized in extraction buffer. The samples were centrifuged at 10,000×g for 10 min to remove insoluble material. Of these supernatant, 20 μl was aspirated for protein determination, and the rest was deproteinized with a 10 kDa molecular weight cutoff spin filter to remove proteins. Intracellular acetyl-CoA levels were measured by using the Acetyl-Coenzyme A Assay Kit (Sigma, MAK039-1KT, USA) following the manufacturer’s protocol. The data were normalized to protein levels, which was measured by using the BCA method. Differences were evaluated by one-way ANOVA followed by Tukey’s test or by independent samples *t* test with SPSS 17.0. 6–8 biological replicates were performed for each treatment.

## Supplementary Information

**Additional file 1: Fig. S1.** The supplementary figures and tables.

**Additional file 2.** RPKM values of all genes and statistical analyses.

**Additional file 3.** PC scores of genes based on AC-PCA analyses.

**Additional file 4.** Aging-related homology genes in locust.

**Additional file 5.** Membership between genes and modules in different networks.

**Additional file 6.** Eigenvalues of modules in different networks.

**Additional file 7.** Hub genes in each network.

**Additional file 8.** The list of 108 aging-related genes.

## Data Availability

The data that support the findings of this study will be openly available in Sequence Read Archive of National Center for Biotechnology Information at https://www.ncbi.nlm.nih.gov/bioproject/PRJNA562411 and Mendeley Data at 10.17632/3crsymb78p.1.

## Abbreviations

RNAi: RNA interference
WGCNA: weighted gene co-expression network analysis
dsRNA: double-strand RNA
RPKM: reads per kilobase per million reads
AC-PCA: adjustment for confounding principal component analysis
DEG: differentially expressed gene
GO: Gene Ontology
KEGG: Kyoto Encyclopedia of Genes and Genomes
TUNEL: TdT-mediated dUTP nick end labeling
TEM: transmission electron microscopy
qPCR: quantitative polymerase chain reaction
D: days after eclosion
FD: flight distance in 1 h
AV: average flight velocity
*LIPT1*: lipoyltransferase 1
*IAP1*: death-associated inhibitor of apoptosis 1
*PGRP-SA*: peptidoglycan recognition protein SA
*PINK1*: PTEN-induced putative kinase 1
