# Supplementary material for: Aging features of the migratory locust at physiological and transcriptional levels
Source: BMC Genomics. 2021 Apr 10;22:257. doi: 10.1186/s12864-021-07585-3 (PMC8037904; doi:10.1186/s12864-021-07585-3)
Supplement: Supplementary file 1 — Additional file 1: Fig. S1. The supplementary figures and tables. [file 12864_2021_7585_MOESM1_ESM.docx]

Supplementary Information for

Aging features of the migratory locust at physiological and transcriptional levels

Siyuan Guo^#^, Pengcheng Yang^#^, Bo Liang, Feng Zhou, Li Hou, Liushu Dong, Le Kang*, Xianhui Wang*

Dr. Xianhui Wang

E-mail: wangxh@ioz.ac.cn

Dr. Le Kang

E-mail: lkang@ioz.ac.cn

**This file includes:**

Figures S1-8

Table S1 and 2.

# Supplementary Information Text

**
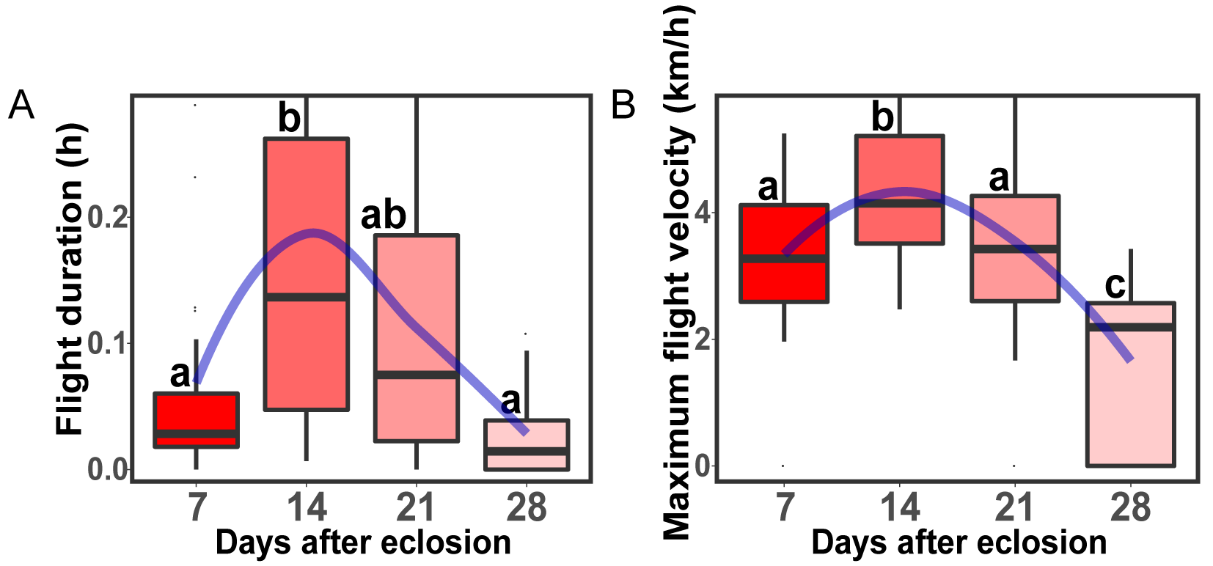
**

**Fig. S1. Flight duration and maximum flight velocity of adult males at different ages**. (**A**) Flight duration and (**B**) maximum flight velocity of adult males at 7, 14, 21, and 28 D. Significant differences are denoted by letters (one-way ANOVA, *P* < 0.05). Blue lines represent the loess curve fitting of flight performance with age.

**
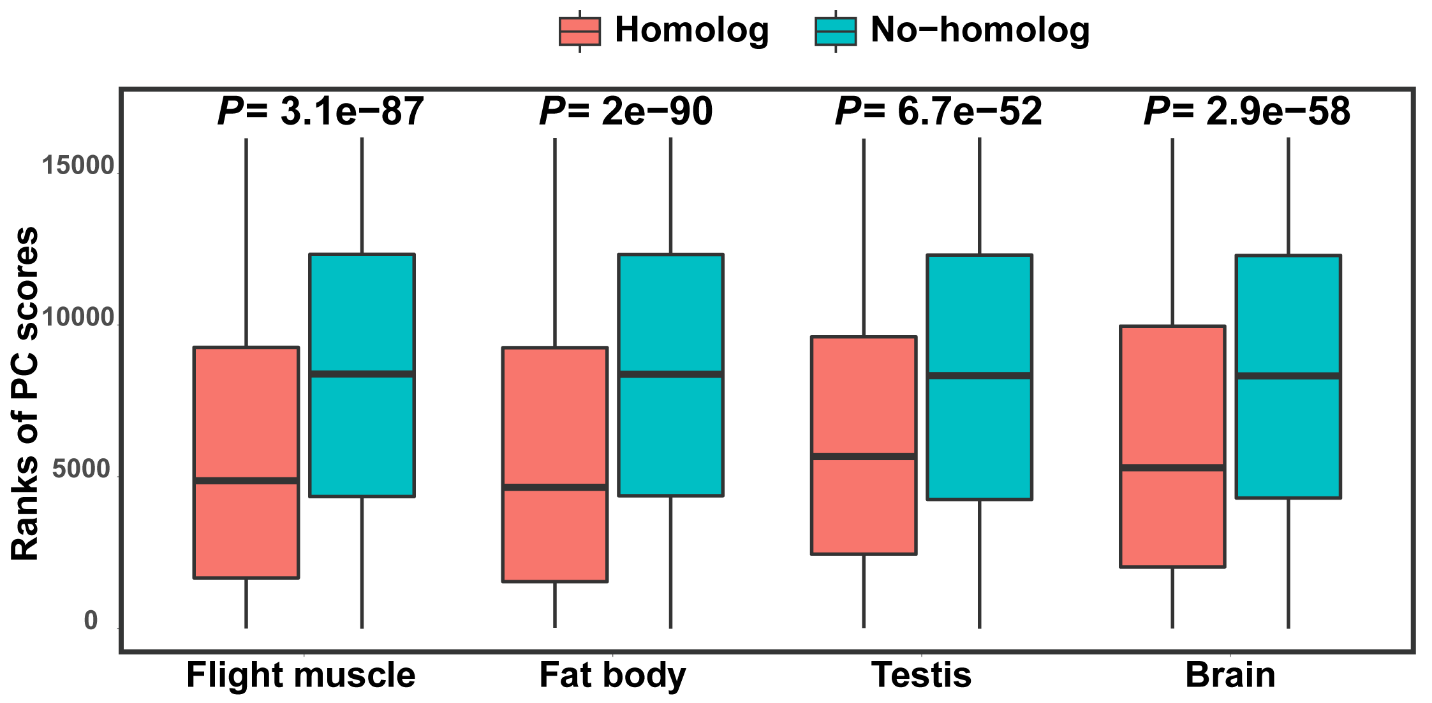
**

**Fig. S2. Similarity of aging-related genes in four studied organs of locusts and model species.** The boxplots show the rank distribution of locust genes with or without identified orthologs in the GenAge database of each organ. The locust genes were ordered on the basis of the absolute values of the corresponding principal component from AC-PCA. Statistical significance was determined via the Mann–Whitney U test.

**
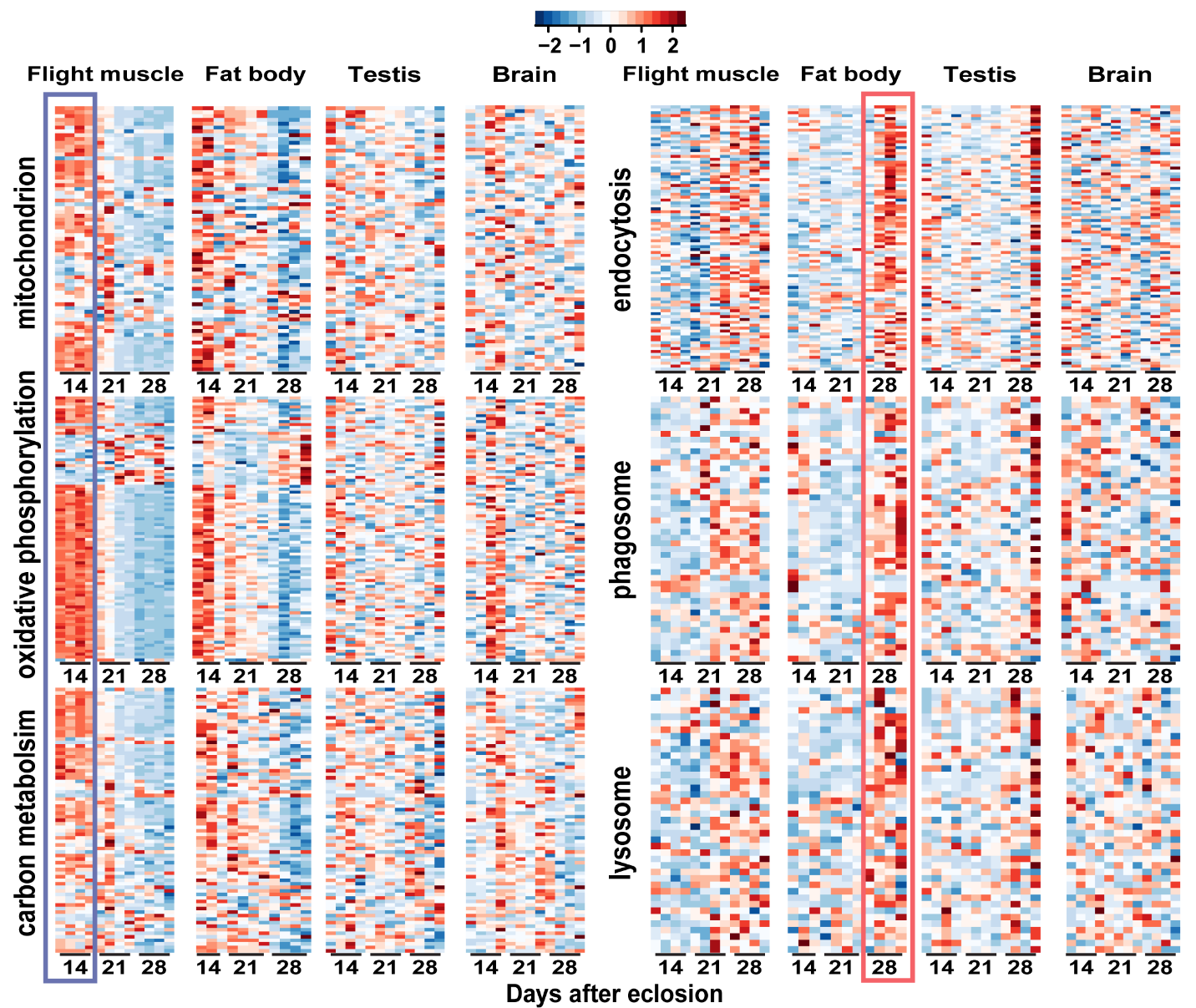
**

**Fig. S3. Heatmap of all genes belonging to the selected terms.** The heatmaps show the relative expression patterns of all genes belonging to selected GO and KEGG terms (i.e., mitochondrion, oxidative phosphorylation, carbon metabolism, endocytosis, phagosome, and lysosome) upon aging across the four organs. The high and low expression levels of each gene is marked in red and blue, respectively.

**
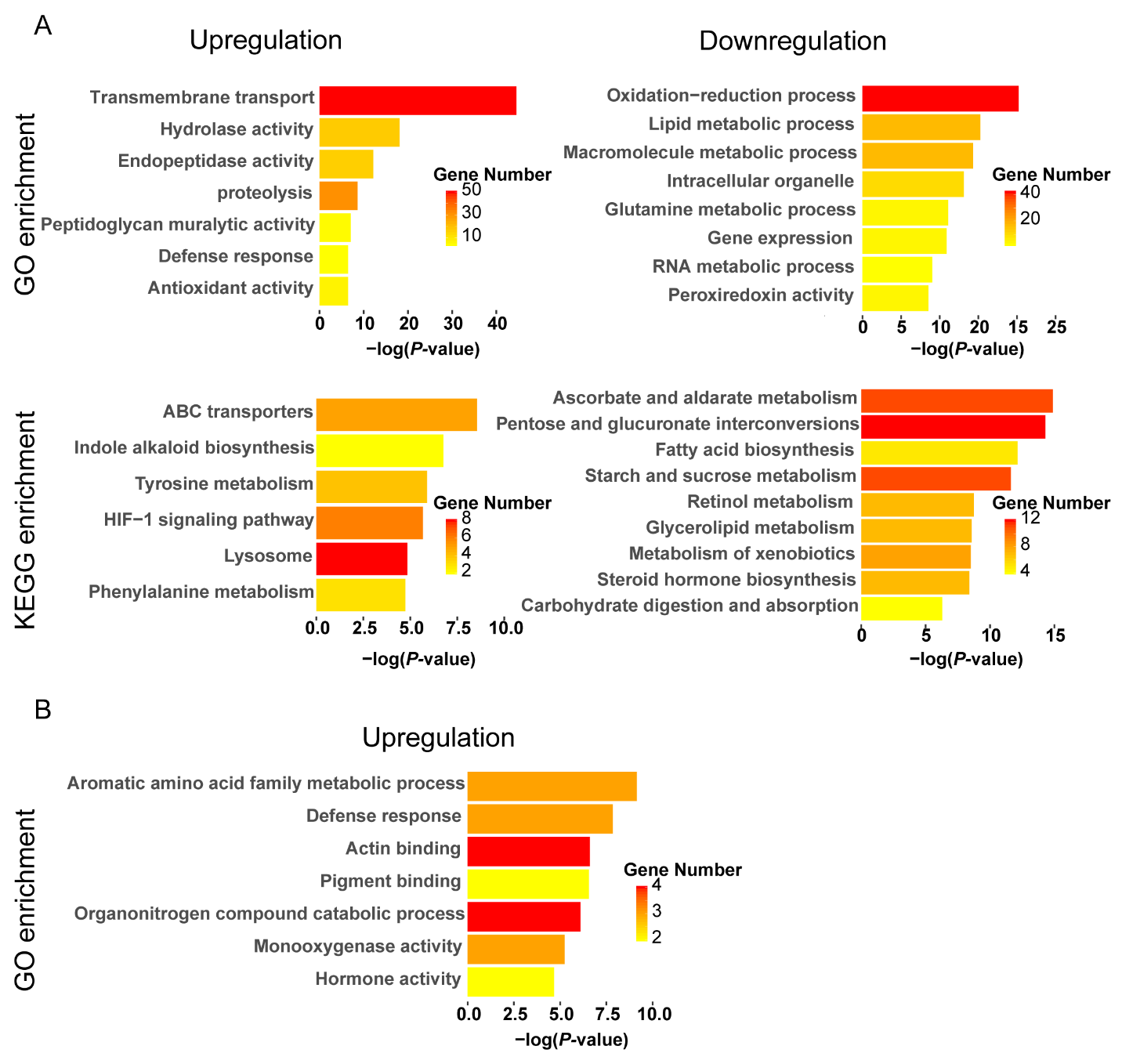
**

**Fig. S4. Enriched terms for the DEGs in fat body and flight muscle.** GO and KEGG enrichment of the DEGs at 21–28 D of fat body (**A**) and upregulated DEGs at 14–21 D of flight muscle (**B**). The length of the bar indicates the *P*-values (a longer bar indicates a more significant term). The bar color indicates the relative number of genes annotated to the term (a deeper red color indicates a higher number).

**
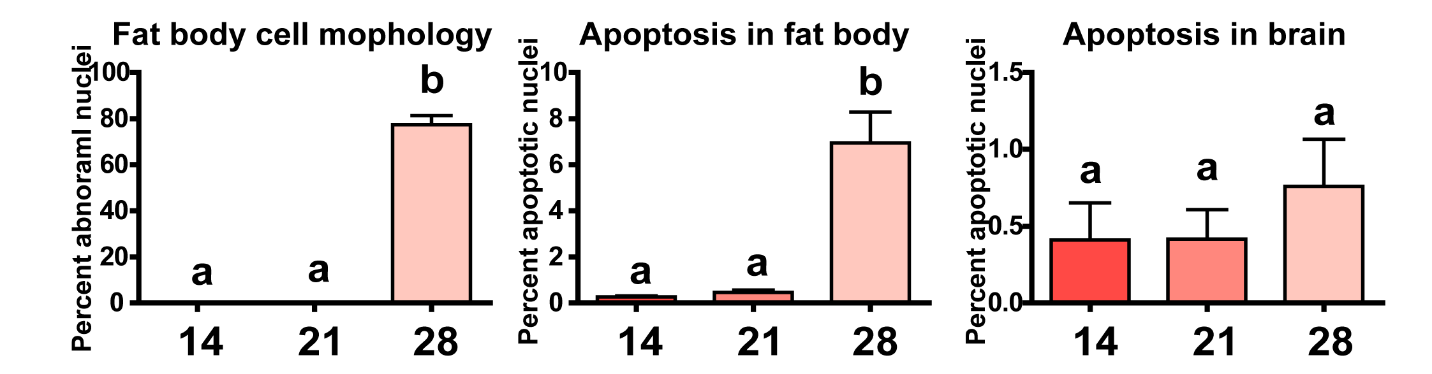
**

**Fig. S5. Percentages of abnormal and apoptotic nuclei in fat body or brain at 14, 21, and 28 D.** The values are expressed as mean ± S.E.M. Significant differences are denoted by different letters (one-way ANOVA, *P* < 0.05).

**
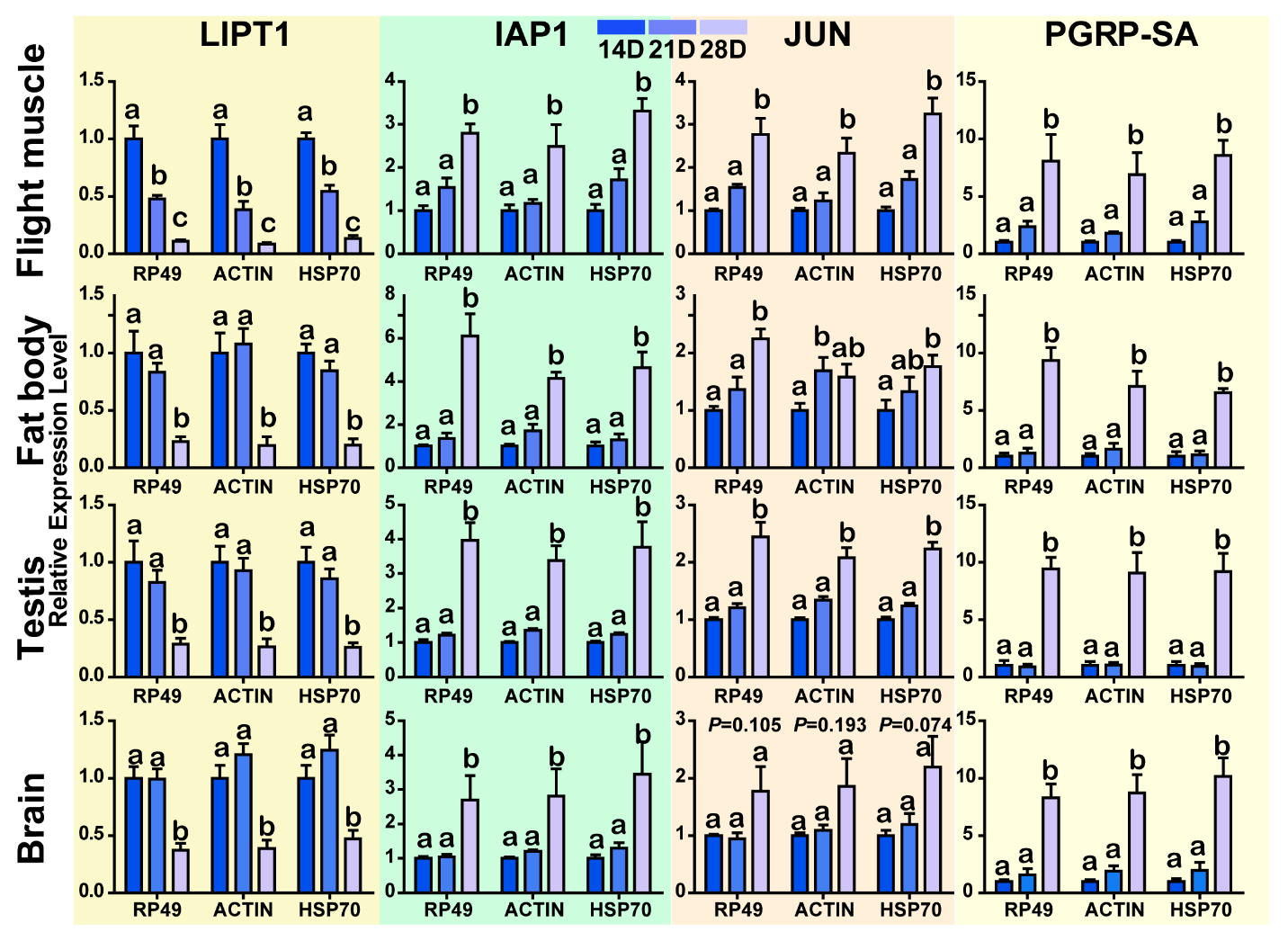
**

**Fig. S6. Assessments of the relative expression levels of studied genes by quantitative polymerase chain reaction (qPCR).** qPCR examination of the expression of selected genes in the four organs of locusts during aging. Reference genes are RP49, ACTIN, and HSP70. Significant differences are revealed using one-way ANOVA, *P* < 0.05.


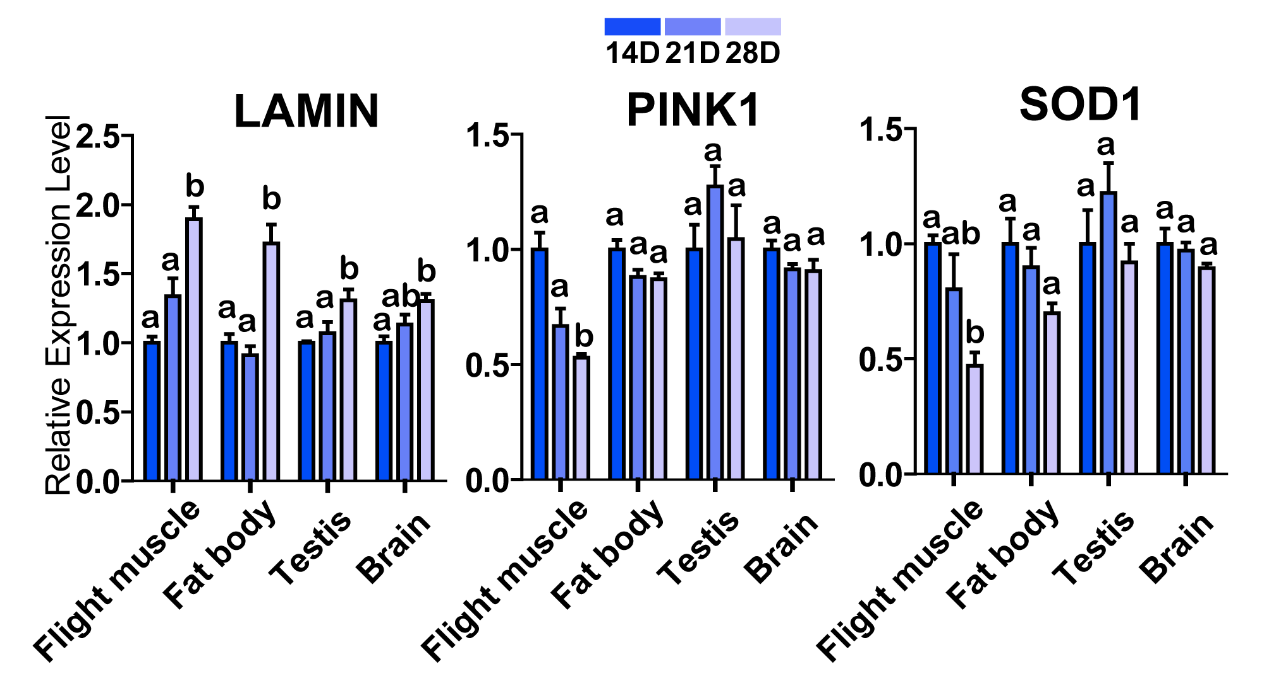


**Fig. S7. Relative mRNA expression levels of three well-known aging genes during aging across the four organs based on transcriptome data.**


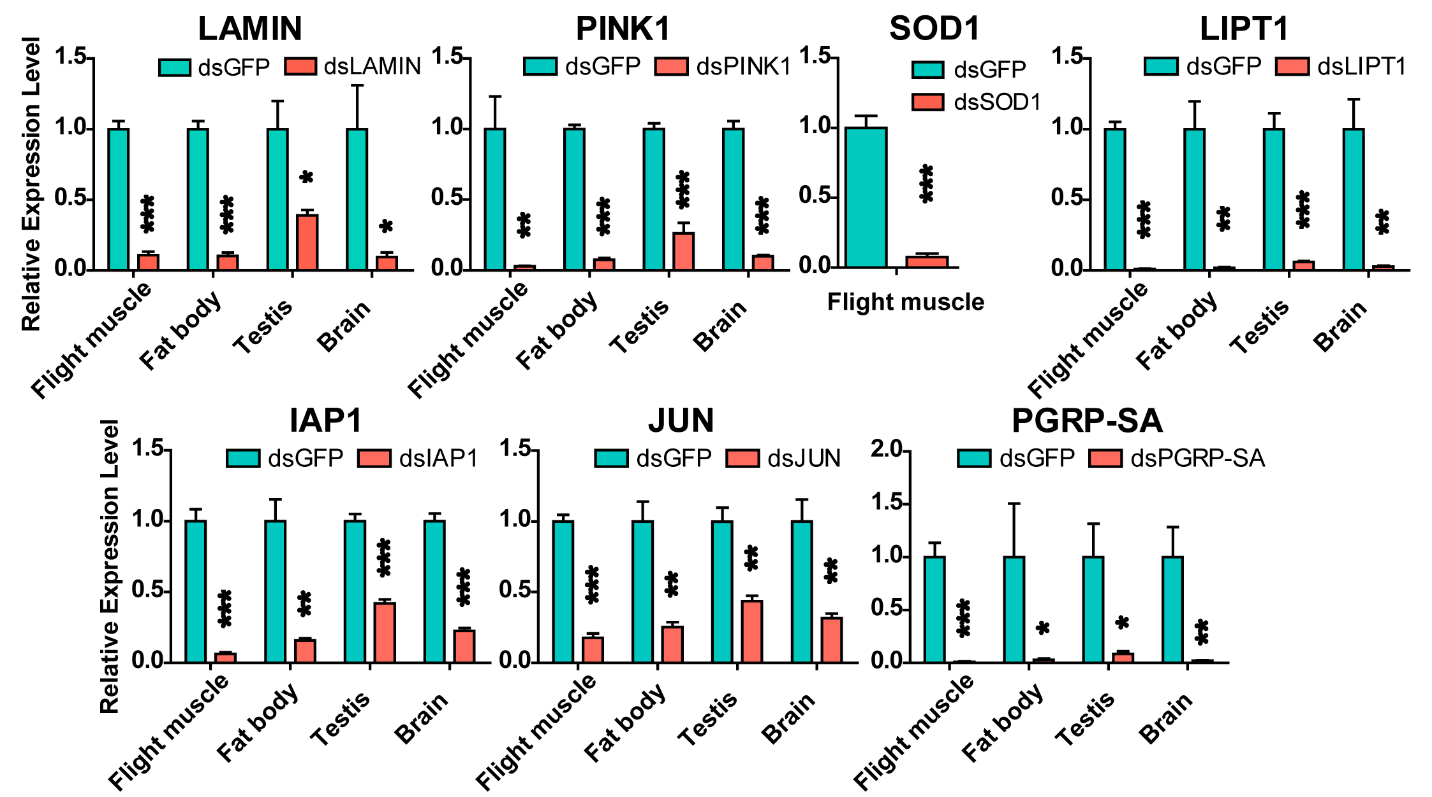
 **Fig. S8. Assessments of the relative expression levels of studied genes by quantitative polymerase chain reaction (qPCR).** qPCR examination of the expression of selected genes in the four organs of locusts 72 h after dsRNA injections. Significant differences are revealed using Student’s *t* test, **P*  <  0.05, ***P*  <  0.01, ****P*  <  0.001.

**Table. S1**

**Statistics for all lifespan experiments described in the current study**

| Targeted genes | *n* | Lifespan starting point, Days after eclosion | Median lifespan | Mean lifespan | Maximum lifespan | Log-rank  *P* value | | Change in median lifespan vs. control, % | | Change in maximal lifespan vs. control, % |
| --- | --- | --- | --- | --- | --- | --- | --- | --- | --- | --- |
| Wild type | 67 | 0 | 22 | 19.8 | 33 |  |  | |  | |
| dsGFP | 27 | 2 | 23 | 19.3 | 30 |  |  | |  | |
| dsLAMIN | 46 | 2 | 6 | 7 | 14 | <0.0001 | 73.9 | | 53.3 | |
| dsGFP | 31 | 2 | 21 | 19.1 | 30 |  |  | |  | |
| dsPINK1 | 29 | 2 | 9 | 10.5 | 23 | <0.0001 | 57.1 | | 23.3 | |
| dsGFP | 37 | 2 | 20.5 | 20.1 | 32 |  |  | |  | |
| dsSOD1 | 31 | 2 | 16 | 16.4 | 28 | 0.0163 | 22.0 | | 12.5 | |
| dsGFP | 32 | 2 | 18.5 | 16.7 | 31 |  |  | |  | |
| dsLIPT1 | 30 | 2 | 9 | 9.1 | 24 | <0.0001 | 51.4 | | 22.6 | |
| dsGFP | 35 | 2 | 20 | 18.0 | 30 |  |  | |  | |
| dsIAP1 | 33 | 2 | 4 | 3.8 | 9 | <0.0001 | 80 | | 70 | |
| dsGFP | 32 | 2 | 18 | 17.1 | 32 |  |  | |  | |
| dsJUN | 30 | 2 | 10.5 | 10.8 | 23 | 0.0002 | 41.7 | | 28.1 | |
| dsGFP | 29 | 2 | 21 | 18.5 | 33 |  |  | |  | |
| dsPGRP  -SA | 29 | 2 | 16 | 14.6 | 26 | 0.0068 | 23.8 | | 21.2 | |

**Table. S2**

**Primers used in the current study.**

| Genes | Primer | Sequences, 5’-3’ | Description |
| --- | --- | --- | --- |
| LAMIN | LmLAMIN-Q-F | CAGTTTTCAAATTCCACC |  |
|  | LmLAMIN-Q-R | TTGTCATACTTTCCCCTA |  |
| PINK1 | LmPINK1-Q-F | CGCCAGACCGAATCAAAACA |  |
|  | LmPINK1-Q-R | CCTCACCACTGACGCACCCC |  |
| SOD1 | LmSOD1-Q-F | GGCACATTTCAACCCTCACAG |  |
|  | LmSOD1-Q-R | TTAACTTTGGCAACTCCATCCC |  |
| LIPT1 | LmLIPT1-Q-F | CTGTCTTACCAGGGAGTTGA |  |
|  | LmLIPT1-Q-R | GTACTTGCTTCTTGGCGTGA |  |
| IAP1 | LmIAP1-QF | TCCACCCAACAACACCAAAC |  |
|  | LmIAP1-QR | CAGATTCCGAACTGTACCTATT | **qPCR** |
| JUN | LmJUN-Q-F | TGAAGCGTAGCCTCACATTG |  |
|  | LmJUN-Q-R | TCCAACTCGGGAGAACCAAT |  |
| PGRP−SA | LmPGRP−SA-Q-F | GGCTACTCGTTCCTGGTGGG |  |
|  | LmPGRP−SA-Q-R | CGATGCCGATGGAGTTCTTGT |  |
| Rp49 | LmRp49-Q-F | CGTAAACCGAAGGGAATTGA |  |
|  | LmRp49-Q-R | GAAGAAACTGCATGGGCAAT |  |
| Actin  Hsp70 | Actin-Q-F  Actin-Q-R  Hsp70-Q-F  Hsp70-Q-R | AAAGAAATCACTGCCCTTGC  ATAGACCCTCCAATCCAAAC  CAGATTGAGAACCGCTTGTG  ACCTTGCCCTGGTGATAGAG |  |
| LAMIN | LmLAMIN-T7-F | TAATACGACTCACTATAGGAGAAGTGAGAACCCGTCGTC |  |
|  | LmLAMIN-T7-R  LmLAMIN-F  LmLAMIN-R | TAATACGACTCACTATAGGTTCCAGCAGTTTCCTGTATG  AGAAGTGAGAACCCGTCGTC  TTCCAGCAGTTTCCTGTATG |  |
| PINK1 | LmPINK1-T7-F | TAATACGACTCACTATAGGGGAAATCCTGGGGCTGTTTG |  |
|  | LmPINK1-T7-R  LmPINK1-F  LmPINK1-R | TAATACGACTCACTATAGGCCGTCTCTGCGGAGTGGTAG  GGAAATCCTGGGGCTGTTTG  CCGTCTCTGCGGAGTGGTAG |  |
| SOD1 | LmSOD1-T7-F | TAATACGACTCACTATAGGATGACTATCAAAGCCGTATGTGTG |  |
|  | LmSOD1-T7-R  LmSOD1-F  LmSOD1-R | TAATACGACTCACTATAGGTGCCTTGGCGATGCCGATC  ATGACTATCAAAGCCGTATGTGTG  TGCCTTGGCGATGCCGATC |  |
| LIPT1 | LmLIPT1-T7-F | TAATACGACTCACTATAGGGAGGCACAGTTTATCACGAC | **dsRNA** |
|  | LmLIPT1-T7-R  LmLIPT1-F  LmLIPT1-R | TAATACGACTCACTATAGGATTTGCTCCTTTCCACCATC  GAGGCACAGTTTATCACGAC  ATTTGCTCCTTTCCACCATC |  |
| IAP1 | LmIAP1- T7-F | TAATACGACTCACTATAGGCTAAATACAGCACGCAGGAG |  |
|  | LmIAP1- T7-R  LmIAP1-F  LmIAP1-R | TAATACGACTCACTATAGGACTTAACGCAGGCAACAATA  CTAAATACAGCACGCAGGAG  ACTTAACGCAGGCAACAATA |  |
| JUN | LmJUN-T7-F | TAATACGACTCACTATAGGCAGCGTGCGGTCAGCATCTA |  |
|  | LmJUN-T7-R  LmJUN-F  LmJUN-R | TAATACGACTCACTATAGGCTGTTGCGTTGCCTCTTCCT  CAGCGTGCGGTCAGCATCTA  CTGTTGCGTTGCCTCTTCCT |  |
| PGRP−SA | LmPGRP−SA-T7-F | TAATACGACTCACTATAGGAAGACGGTGGAGTACATCATAAA |  |
|  | LmPGRP−SA-T7-R  LmPGRP-SA-F  LmPGRP-SA-R | TAATACGACTCACTATAGGACGTGGAATGAGTTGAGTGTTAG  AAGACGGTGGAGTACATCATAAA  ACGTGGAATGAGTTGAGTGTTAG |  |
| GFP | GFP-T7-F | TAATACGACTCACTATAGGCACAAGTTCAGCGTGTCCG |  |
|  | GFP-T7-R | TAATACGACTCACTATAGGGTTCACCTTGATGCCGTTC |  |
|  | GFP-F | CACAAGTTCAGCGTGTCCG |  |
|  | GFP-R | GTTCACCTTGATGCCGTTC |  |
